# *VvU2A′*-mediated circRNA biogenesis confers salt tolerance in grapevine via the *VvcircHMA1-*VvmiR167b-*VvARF6* pathway

**DOI:** 10.1093/hr/uhaf355

**Published:** 2025-12-22

**Authors:** Zhen Gao, Le Zheng, Yeqi Li, Jing Li, Yuanpeng Du

**Affiliations:** Shandong Key Laboratory of Fruit and Vegetable Germplasm Innovation and Utilization; College of Horticulture Science and Engineering, Shandong Agricultural University, Tai'an, Shandong, China; Shandong Key Laboratory of Fruit and Vegetable Germplasm Innovation and Utilization; College of Horticulture Science and Engineering, Shandong Agricultural University, Tai'an, Shandong, China; Shandong Key Laboratory of Fruit and Vegetable Germplasm Innovation and Utilization; College of Horticulture Science and Engineering, Shandong Agricultural University, Tai'an, Shandong, China; Shandong Key Laboratory of Fruit and Vegetable Germplasm Innovation and Utilization; College of Horticulture Science and Engineering, Shandong Agricultural University, Tai'an, Shandong, China

## Abstract

Circular RNAs (circRNAs) play important roles in plant stress responses, yet their dynamic regulation during stress remains unclear. This study elucidates a molecular mechanism whereby the grapevine U2 snRNP core component *VvU2A′* enhances salt tolerance through a circRNA-mediated post-transcriptional network. We found that *VvU2A′* expression is induced by salt stress and positively regulates salt tolerance in grapevine. CircRNA sequencing revealed 497 *VvU2A′*-regulated differentially expressed circRNAs, including downregulated *VvcircHMA1*. Mechanistic investigation revealed that *VvcircHMA1* acts as a competitive endogenous RNA by sequestering VvmiR167b, thereby attenuating its cleavage activity on the target mRNA *VvARF6*. Functional analyses revealed that both *VvcircHMA1* and *VvARF6* negatively regulate salt tolerance, while VvmiR167b positively regulates it. Collectively, our study reveals a novel mechanism by which the splicing factor *VvU2A′* enhances salt stress response through the *VvcircHMA1*-VvmiR167b-*VvARF6* cascade, providing promising molecular targets for breeding salt-resistant grapevines.

## Introduction

Abiotic stresses severely inhibit crop growth and development, impairing both yield and quality traits, which have been emerged as a critical challenge to global agricultural productivity. Precise pre-mRNA splicing, catalyzed by the dynamic spliceosome complex, is essential for eukaryotic gene regulation [1, 2]. The canonical spliceosome comprises five small nuclear ribonucleoproteins (snRNPs): U1, U2, U4/U6, and U5 [3]. Accumulating evidence demonstrates that spliceosomal components regulate plant stress responses by modulating pre-mRNA splicing. For example, the *Arabidopsis* core spliceosomal protein SmEb regulates salt tolerance by altering *RCD1* splicing [4], and U1A controls salt resistance via modulating *ACO1* alternative splicing [5]. U2A′, a key RNA-binding protein within the U2 snRNP, is indispensable for pre-mRNA splicing [6]. Recent studies show that U2A′ deficiency in *C. deneoformans* JEC21 reduces tolerance to temperature extremes, osmotic stress, and oxidative stress [7]. However, the function and molecular mechanisms of U2A′ in plants remain unexplored.

Circular RNAs (circRNAs), a prominent class of noncoding RNA molecules, are ubiquitous across organisms. They are generated from back-splicing of pre-mRNAs, forming covalently closed loop structures with neither 5′ oop struolarity nor polyadenylated tail [8, 9]. Since the landmark discovery in 2013 that circRNAs can function as ‘micro-RNA sponges’ to sequester miRNAs and thereby modulate the expression of downstream target genes [10, 11], research on plant circRNAs has gained increasing momentum. Accumulating evidence indicates that plant circRNAs are responsive to a variety of environmental stresses, including drought, nutrient deficiency, temperature fluctuations, and salinity [11, 12]. Moreover, functional characterization of plant circRNAs is expanding [12, 13]. For example, circANK in rice negatively regulates resistance to bacterial blight [14], and our previous work demonstrated that *Vv-circSIZ1* and *Vv-circABH* positively regulate salt tolerance in grapevine [15, 16]. The biogenesis of circRNAs is governed by the interplay of cis-regulatory elements and trans-acting factors. While mechanisms underlying circRNA formation are relatively well characterized in animals, implicating intronic ALU repeats and specific RNA-binding proteins as key players [17, 18], the understanding of circRNA biogenesis in plants remains comparatively limited. Bioinformatic analyses reveal that the canonical GT/AG splice signals predominantly flank circRNAs in cucumber, rice, and grapevine [15, 19, 20], and research in *Arabidopsis* indicates that circRNAs accumulate significantly in splicing-related mutants, such as cbp80, c2h2, and flk [21]. Complementing this, our work in grapevine established that circRNA biogenesis is dependent on the spliceosome, with the majority of U1 and U2 snRNP-specific proteins acting as negative regulators of circRNA expression [15]. Collectively, these findings underscore the crucial role of splicing factors in modulating circRNA production in plants. Nevertheless, a fundamental question remains unresolved: how do splicing factors in plants respond to environmental stresses to modulate the biogenesis of specific circRNAs, thereby regulating stress resistance?

Current understanding of circRNA functional mechanisms predominantly centers on their role as competitive endogenous RNAs (ceRNAs), which regulate target gene expression by interacting with microRNAs (miRNAs) [22]. Among the most abundant short noncoding RNAs in plants, miRNAs typically function by either cleaving target transcripts or repressing their translation [23]. The miR167 family represents a highly conserved group of miRNAs integral to plant development and stress responses [24]. Research demonstrates that miR167 modulates key physiological processes—including the development of vegetative and reproductive organs, flowering time control, and stress adaptation—primarily through regulating its core target genes *ARF6*, *ARF8*, and *IAR3* [24]. For instance, in *Arabidopsis thaliana*, miR167 governs the development of pistils, stamens, and petals and controls somatic embryogenesis by regulating *ARF6/ARF8* [25, 26]. Under cold stress, strawberry miR167 delays fruit senescence by modulating *ARF8* expression and thereby influencing hormone biosynthesis [ 27]. Heterologous overexpression of grape miR167 in *Arabidopsis* significantly enhances thermotolerance [28]. Furthermore, *Arabidopsis* miR167 positively regulates resistance to *Pseudomonas syringae* via its targets *ARF6* and *ARF8* [29]. Collectively, these findings underscore the critical importance of miR167 targeting of ARF genes in regulating diverse aspects of plant development and biotic stress responses. However, its involvement in salt stress responses remains.

Salt stress constitutively inhibits root development in grapevine and severely impedes shoot growth, ultimately leading to plant mortality [30]. Current research on grapevine salt stress responses has predominantly focuses on transcriptional regulation. However, the post-transcriptional regulatory mechanisms, particularly those involving spliceosomal components and circRNA-mediated networks, remain poorly understood. To address this knowledge gap, this study investigates whether *VvU2A′*, a core component of the U2 snRNP spliceosomal complex in grapevine, modulates grapevine salt tolerance through regulating the expression of specific circRNAs. To our knowledge, this represents the first evidence in plants linking a spliceosomal factor to stress resistance via the regulation of circRNA biogenesis. Elucidating the *VvU2A′***-**circRNA**-**miRNA regulatory pathway will significantly advance our mechanistic understanding of spliceosomal functions in plants and the roles of circRNA-mediated post-transcriptional networks in abiotic stress adaptation.

## Results

### Response of U2snRNP core component expression to salt stress

**Figure 1:**
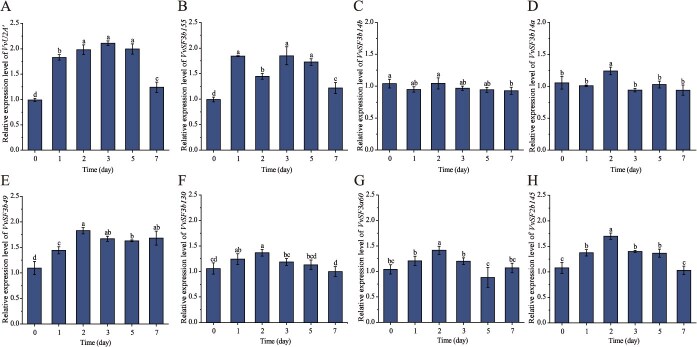
Transcriptional responses of U2 snRNP core component genes to salt stress in grapevine. Grapevine calli were treated with 100 mM NaCl. Relative mRNA levels were determined by RT-qPCR. Data represent means ± SD of three independent biological replicates. Different letters above the bars indicate significantly different values (*P* < 0.05) calculated using one-way ANOVA, followed by Duncan's multiple range test.

Using *A. thaliana* U2 snRNP protein sequences as queries, we performed BLASTP analysis against the grapevine genome and identified eight core U2 snRNP proteins. To screen for U2 snRNP members involved in salt stress response, grapevine calli were subjected to salt treatment followed by RT-qPCR analysis (Fig. 1). Results revealed that seven genes exhibited transient upregulation followed by downregulation, while *SF3b14b* expression remained uninduced. Notably, *VvU2A′* showed the highest induction among all genes throughout the 1- to 5-day salt treatment period, thus selected for further investigation.

### Molecular characteristics and subcellular localization of *VvU2A*′

**Figure 2:**
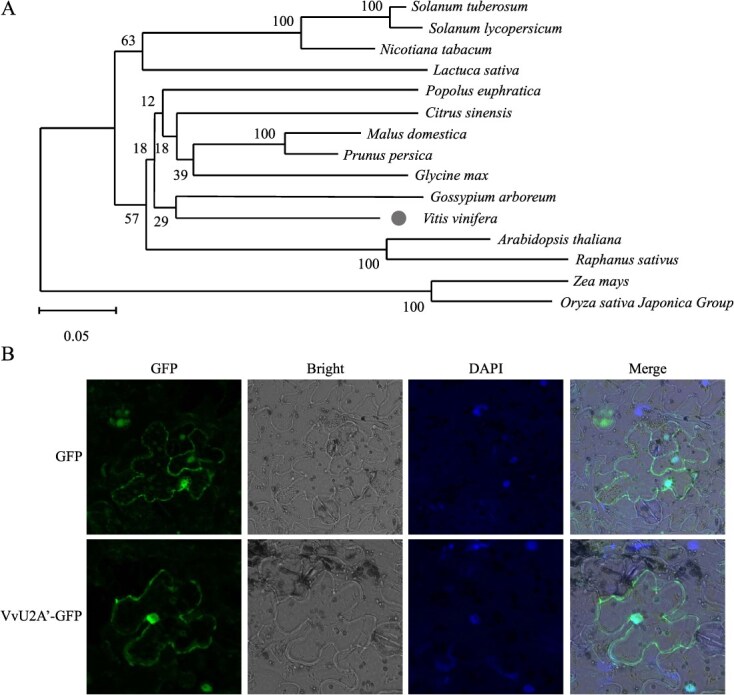
Phylogenetic analysis and subcellular localization of grapevine VvU2A′. (A) Phylogenetic tree of U2A′ proteins constructed using the neighbor-joining method. Bootstrap values from 1000 replicates are indicated at the branch nodes. (B) Subcellular localization of the VvU2A′–GFP fusion protein in *N. benthamiana* leaf epidermal cells. The fusion protein was transiently expressed via *A. tumefaciens*-mediated infiltration. GFP fluorescence (green), nuclei stained with DAPI (blue), merged image, and bright-field view are shown.

Phylogenetic analysis revealed high conservation of U2A′ proteins across species, with *Vitis vinifera* U2A′ showing closest homology to *Glycine soja* orthologs (Fig. 2A). Transient expression in *Nicotiana benthamiana* leaves via *Agrobacterium*-mediated transformation *N. benthamiana* revealed nuclear and plasma membrane localization of *VvU2A′* (Fig. 2B).

Secondary structure prediction indicated that *VvU2A′* is predominantly composed of α-helices and random coils (>90% combined), with minimal β-strand content (Fig. S1A). Tertiary structure modeling based on these predictions revealed a compact globular fold (Fig. S1B). Conserved domain analysis identified a canonical leucine-rich repeat motif in *VvU2A′* (Fig. S1C). Analysis of the *VvU2A′* promoter region revealed the presence of diverse cis-acting elements, including those responsive to gibberellin, light, salicylic acid, and abscisic acid (Fig. S1D).

### *VvU2A′* positively regulates plant salt tolerance

**Figure 3:**
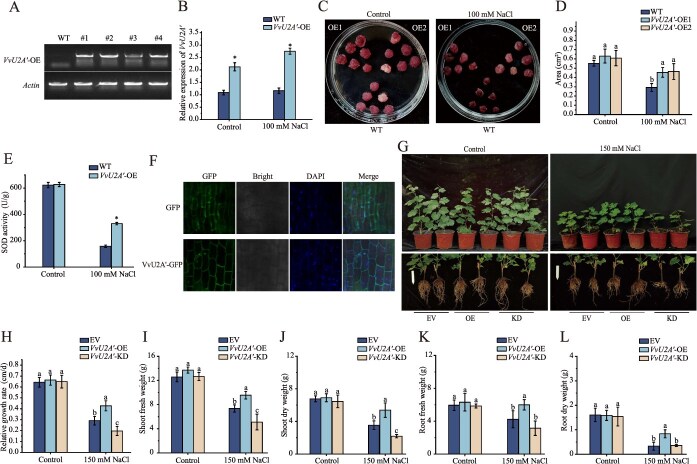
*VvU2A.* positively regulates salt tolerance in grapevine. (A) Validation of overexpression vector expression in *VvU2A*-OE transgenic calli. Total RNA was extracted from calli and analyzed by RT-PCR using a forward primer specific to *VvU2A.* and a reverse primer designed to target the expression vector. (B) RT-qPCR analysis of *VvU2A.* expression levels in *VvU2A*-OE transgenic calli. (C–E) Phenotype (C), relative growth surface area (D), and superoxide dismutase (SOD) activity (E) of *VvU2A*-OE calli grown on medium containing 100 mM NaCl. (F) GFP fluorescence detection in transgenic grapevine seedling roots generated via *A. rhizogenes*–mediated transformation. (G–L) Salt tolerance analysis of composite grapevine seedlings with transgenic roots overexpressing (*VvU2A.*-OE) or silencing (*VvU2A.*-KD) *VvU2A′.* (G) More than 30 independent transgenic lines were irrigated with 150 mM NaCl for 21 days and typical lines are shown. The following parameters were measured: relative shoot growth rate (H), shoot fresh weight (I), shoot dry weight (J), root fresh weight (K), and root dry weight (L). Data represent means ± SD of three independent biological replicates. Asterisks above the bars indicate significant differences determined by Student's *t* test (*P* < 0.05). Different letters above the bars indicate significantly different values (*P* < 0.05) calculated using one-way ANOVA, followed by Duncan's multiple range test.

To investigate the biological function of *VvU2A′,* we generated stable overexpression lines (*VvU2A′*-OE) in grape calli via *Agrobacterium tumefaciens***-**mediated transformation. Transgenic lines were verified by RT-PCR using both gene-specific and vector-specific primers. All four independent transgenic lines showed positive amplification, while no PCR product was detected in wild-type (WT) calli, confirming successful integration of the *VvU2A′*-OE construct into the grape genome (Fig. 3A). RT-qPCR analysis confirmed significantly higher *VvU2A′* expression in *VvU2A′*-OE calli compared to wild-type (WT), with a further increase following salt stress treatment (Fig. 3B). Under normal growth conditions, no significant differences in growth status were observed between WT and *VvU2A′*-OE calli. However, upon salt stress treatment, the *VvU2A′*-OE calli exhibited significantly faster growth rates than the WT calli (Fig. 3C and D). Consistent with this enhanced growth phenotype, superoxide dismutase (SOD) activity was significantly higher in salt-stressed *VvU2A′*-OE calli than in WT (Fig. 3E).

To further validate the function in whole plants, we employed an *A. rhizogenes***-**mediated hairy root transformation system to generate grape plants with transgenic roots. Fluorescence detection confirmed successful transgene expression in the roots (Fig. 3F). Under normal conditions, transgenic and control plants showed no significant growth differences. However, under salt stress, grape plants carrying *VvU2A′*-OE hairy roots exhibited significantly better overall growth than empty vector (EV) control plants. This was characterized by a significantly increased relative shoot growth rate and significantly higher shoot and root biomass (Fig. 3G–L). Conversely, plants carrying *VvU2A′* knockdown (*VvU2A′*-KD) hairy roots displayed more severe growth inhibition under stress. Analysis of physiological and biochemical parameters under salt stress revealed that compared to EV controls, *VvU2A′*-OE plants showed significantly increased activities of catalase (CAT), peroxidase (POD), SOD, and root activity (Fig. S2A–D). Conversely, root malondialdehyde (MDA) content and relative electrical conductivity (REC) were significantly reduced (Fig. S2E and F). Additionally, leaf photosynthetic pigment contents (chlorophyll a, b, carotenoids) and maximal photochemical efficiency (F_v_/F_m_) were significantly higher (Fig. S2G–K). Furthermore, sodium ion (Na^+^) content was significantly lower, while potassium ion (K^+^) content was significantly higher, in roots, stems, and leaves, leading to a significantly reduced Na^+^/K^+^ ratio (Fig. S2L–T). Conversely, *VvU2A′*-KD plants exhibited opposite trends in these parameters compared to *VvU2A′*-OE plants. These results collectively indicate that *VvU2A′* positively regulates salt tolerance in grapevine.

To extend these findings to model species, we generated homozygous *VvU2A′*-OE lines in *A. thaliana* (Fig. S3A). Under salt stress, *VvU2A′*-OE *Arabidopsis* lines exhibited significantly higher germination rates than WT (Fig. S3B–D). Growth assays on MS medium supplemented with salt demonstrated that *VvU2A′*-OE seedlings had significantly longer primary roots and greater fresh weight than WT under stress (Fig. S3E–G). Phenotypic observation of soil-grown seedlings subjected to salt stress revealed less damage and significantly reduced leaf yellowing in *VvU2A′*-OE plants compared to WT (Fig. S3H). Physiological measurements confirmed that salt-stressed *VvU2A′*-OE plants had significantly higher root activity and chlorophyll content, but significantly lower leaf MDA content than WT (Fig. S3I–K). Furthermore, 3,3*′*-diaminobenzidine (DAB) and nitroblue tetrazolium (NBT) staining showed less intense staining in *VvU2A′*-OE leaves than in WT, indicating reduced accumulation of H₂O₂ and O₂^−^, respectively, under salt stress (Fig. S3L). Together, these data demonstrate that *VvU2A′* overexpression enhances salt tolerance in *Arabidopsis*.

### *VvU2A′* overexpression alters circRNA expression profiles in grapevine under salt stress

**Figure 4:**
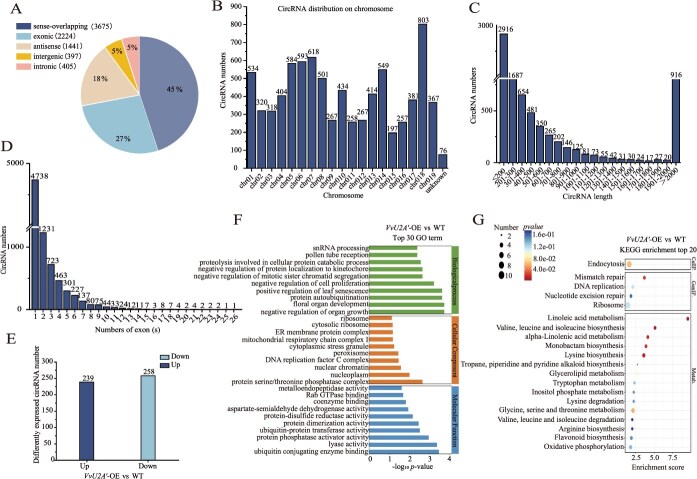
Identification and characterization of circular RNAs regulated by *VvU2A′*. (A) Classification of circRNAs based on genomic origins or positional relationships with adjacent linear transcripts (parental genes). (B) Genomic distribution of circRNAs across grapevine chromosomes. (C) Length distribution of identified circRNAs. (D) Distribution of the number of exons per circRNA. (E) Number of differentially expressed circRNAs (DECs) identified in *VvU2A′*-OE calli vs. control under salt stress. (F) Gene ontology (GO) enrichment analysis of DEC parental genes. (G) Kyoto Encyclopedia of Genes and Genomes (KEGG) pathway enrichment analysis of parental genes for DECs.

CircRNA sequencing was performed on salt-stressed *VvU2A′*-OE and WT grape calli, identifying 11 402 circRNAs in total. Among these, sense-overlapping circRNAs represented the predominant type (45%), followed by exonic (27%) and antisense types (18%), with intronic (5%) and intergenic (5%) being less abundant (Fig. 4A). The circRNAs were distributed across all grape chromosomes (Fig. 4B), with most circRNAs were shorter than 300 bp and predominantly derived from one or two exons (Fig. 4C and D).

Comparative analysis using the thresholds of |fold-change| > 2 and *P*-value <0.05 identified 497 differentially expressed circRNAs (DECs) between salt-stressed *VvU2A′*-OE and WT calli, comprising 239 upregulated and 258 downregulated circRNAs (Fig. 4E). Gene ontology (GO) enrichment of DEC parental genes revealed significant terms, including negative regulation of organ growth and floral organ development in biological process; protein serine/threonine phosphatase complex and nucleoplasm in cellular component; ubiquitin conjugating enzyme binding and lyase activity in molecular function (Fig. 4F). Kyoto Encyclopedia of Genes and Genomes (KEGG) enrichment showed DEC parental genes significantly associated with valine/leucine/isoleucine biosynthesis, linoleic acid metabolism, and α-linolenic acid metabolism pathways (Fig. 4G).

### *VvcircHMA1* negatively regulates salt tolerance

**Figure 5:**
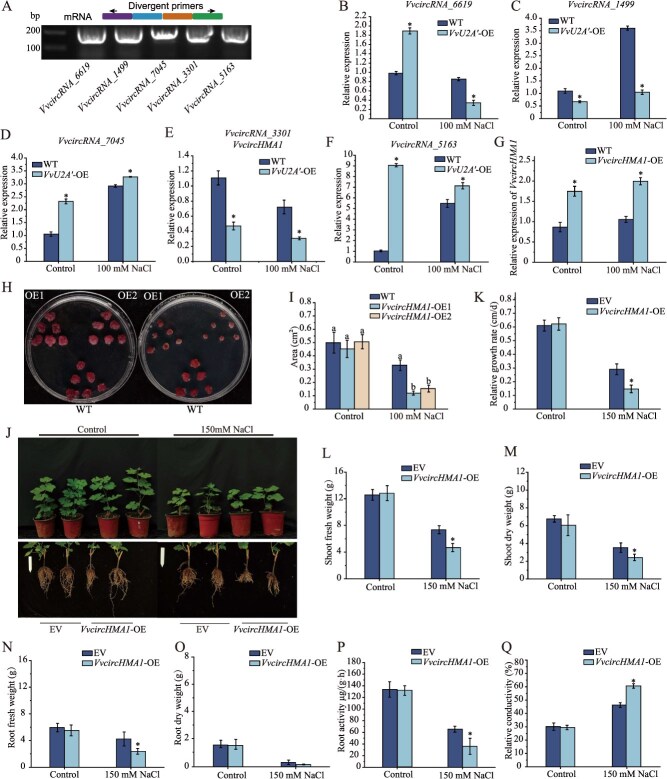
*VvcircHMA1* negatively regulates salt tolerance in grapevine. (A) Detection of candidate circRNAs. Upper panel, a model showing the divergent primers used to amplify the circRNAs. Lower panel, detection of circRNAs in grapevine by RT-PCR. (B–F) RT-qPCR analysis of five circRNAs in wild-type (WT) and *VvU2A′*-OE calli. (G) RT-qPCR analysis of *VvcircHMA1* expression in WT and *VvcircHMA1*-OE calli. (H and I) Salt tolerance comparison between WT and *VvcircHMA1*-OE calli. (H) Phenotype, (I) Relative growth area. (J–Q) Salt tolerance comparison between empty vector (EV) and *VvcircHMA1*-overexpressing grapevine potted seedlings. Roots overexpressing *VvcircHMA1* were generated using an *A. rhizogenes*–mediated hairy root transformation system. More than 30 independent transgenic lines were irrigated with 150 mM NaCl for 21 days and typical lines are shown (J). The following parameters were measured: relative shoot growth rate (K), shoot fresh weight (L), shoot dry weight (M), root fresh weight (N), root dry weight (O), root activity (P), and relative root electrolyte conductivity (Q). Data represent mean ± SD (*n* = 3). Asterisks above the bars indicate significant differences determined by Student's *t* test (*P* < 0.05). Different letters above the bars indicate significantly different values (*P* < 0.05) calculated using one-way ANOVA, followed by Duncan's multiple range test.

Based on functional investigations of circRNA parental genes, eleven candidate circRNAs were selected for RT-PCR validation. Among these, five were successfully confirmed to harbor back-splice junctions (Figs 5A and S4). Subsequent RT-qPCR analysis revealed diverse expression patterns for these five circRNAs in WT and *VvU2A′*OE grape calli under salt stress (Fig. 5B–F). Notably, the expression level of *VvcircRNA_3301* (designated *VvcircHMA1* based on its parental gene) was significantly downregulated under salt stress compared to control conditions in both WT and *VvU2A′*-OE calli. Furthermore, *VvcircHMA1* expression was significantly lower in *VvU2A′*-OE calli than in WT calli (Fig. 5E). This finding aligns with our prior finding that spliceosomal components negatively regulate circRNA biogenesis [15], prompting us to select *VvcircHMA1* for functional characterization.

Transforming grape calli with a *VvcircHMA1* overexpression vector generated *VvcircHMA1*-OE lines, confirmed by RT-qPCR to have significantly elevated *VvcircHMA1* expression (Fig. 5G). While no growth differences were observed under normal conditions, salt-stressed *VvcircHMA1*-OE calli exhibited slower growth rates and significantly smaller basal surface area than WT (Fig. 5H and I). Using an *A. rhizogenes***-**mediated hairy root transformation system, we generated grape plants overexpressing *VvcircHMA1* specifically in their roots. Under salt stress, plants harboring *VvcircHMA1*-OE hairy roots displayed reduced overall growth vigor compared to EV plants (Fig. 5J). These plants also showed significantly lower relative shoot growth rates and shoot weights (Fig. 5K–M), significantly reduced root weights and root activity (Fig. 5N–P), and a significantly increased root REC (Fig. 5Q). Collectively, these results demonstrate that *VvcircHMA1* overexpression compromises salt stress tolerance in grape plants.

Consistently, transgenic *N. benthamiana* plants overexpressing *VvcircHMA1* also exhibited reduced salt tolerance. Under salt stress, the germination rate of *VvcircHMA1*-OE transgenic lines was significantly lower than that of WT *N. benthamiana* (Fig. S5A and B). Growth assays on MS medium revealed that salt-stressed *VvcircHMA1*-OE seedlings had significantly shorter primary roots and lower fresh weights than WT seedlings (Fig. S5C–E). Similarly, soil-grown *VvcircHMA1*-OE *N. benthamiana* plants subjected to salt stress were significantly shorter and lighter than WT plants (Fig. S5F–I). Furthermore, *VvcircHMA1*-OE roots accumulated significantly higher levels of MDA while exhibiting significantly reduced root activity and chlorophyll content (Fig. S5J–L). These data confirm that *VvcircHMA1* overexpression reduces salt stress tolerance in *N. benthamiana*.

### *VvcircHMA1* competitively binds VvmiR167b

**Figure 6:**
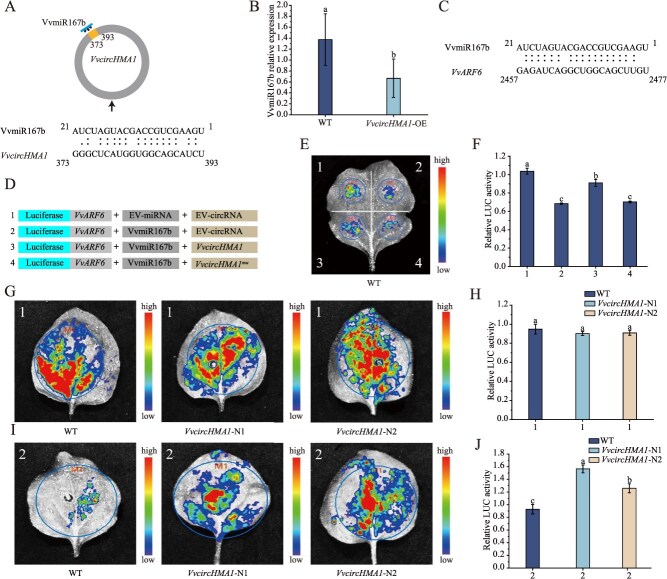
Validation of molecular interaction mechanisms among *VvcircHMA1*, VvmiR167b, and *VvARF6*. (A) Predicted binding site for VvmiR167b on *VvcircHMA1* using psRNATarget. (B) RT-qPCR analysis of VvmiR167b expression in wild-type (WT) and *VvcircHMA1-OE* grapevine calli*. VvActin* as internal control. (C) Predicted binding site for VvmiR167b on *VvARF6.* (D–J) Luciferase reporter assays detecting interactions between *VvcircHMA1*, VvmiR167b and *VvARF6*. (D) Schematic of vector combinations co-infiltrated into *N. benthamiana* leaves. Luciferase activity recorded 3 days after infiltration in leaves of WT or *VvcircHMA1*-OE transgenic *N. benthamiana*. *VvcircHMA1^mu^* represents mutation of VvmiR167b binding sites on *VvcircHMA1*. Different letters above the bars indicate significantly different values (*P* < 0.05) calculated using one-way ANOVA, followed by Duncan's multiple range test.

Fluorescence in situ hybridization (FISH) analysis of *VvcircHMA1*-OE *N. benthamiana* plants demonstrated distinct cytoplasmic localization of *VvcircHMA1*, supporting its potential function in post-transcriptional regulation (Fig. S6). Using psRNATarget software, we identified a putative binding site for VvmiR167b within *VvcircHMA1* (Fig. 6A). Consistent with a potential interaction, VvmiR167b expression was significantly decreased in *VvcircHMA1*-OE plants compared to the WT (Fig. 6B). miR167 is well established for cleaving its target genes *ARF6* and *ARF8* [25]. Sequence analysis confirmed the presence of a VvmiR167b cleavage site within *VvARF6* (Fig. 6C). To validate functional targeting, we performed dual-luciferase reporter assays in *N. benthamiana* leaves. Co-transfection of VvmiR167b with *VvARF6*-LUC significantly reduced luciferase activity compared to transfection with *VvARF6*-LUC alone (Fig. 6D–F) in WT leaves, demonstrating that VvmiR167b cleaves *VvARF6*. Importantly, co-transfection of wild-type *VvcircHMA1* partially restored *VvARF6*-LUC activity, whereas the *VvcircHMA1^mu^* variant (mutated in the VvmiR167b binding site) failed to exert this reversal effect (Fig. 6E and F).

Furthermore, when VvmiR167b and *VvARF6*-LUC were co-transfected into leaves of *N. benthamiana* stably overexpressing *VvcircHMA1*, luciferase activity was significantly higher than that observed in WT leaves subjected to the same co-transfection (Fig. 6I and J). Collectively, these results demonstrate that *VvcircHMA1* binds VvmiR167b, thereby attenuating VvmiR167b-mediated cleavage of *VvARF6*, acting as a competing endogenous RNA.

### VvmiR167b and *VvARF6* confer opposite roles in salt tolerance

**Figure 7:**
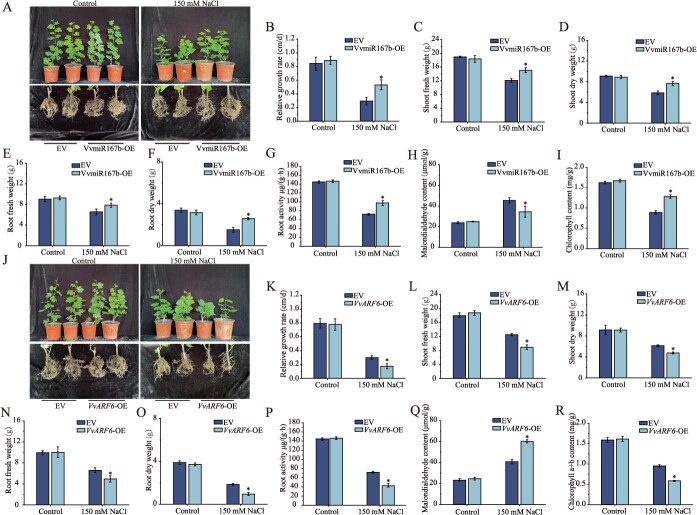
Functional analysis of VvmiR167b and *VvARF6* in salt tolerance of grapevine. Transgenic grapevine plants overexpressing VvmiR167b or *VvARF6* specifically in roots were generated using an *A. rhizogenes*–mediated hairy root transformation system. (A–I) Salt stress response of empty vector (EV) control versus VvmiR167b-overexpressing (OE) lines. (J–R) Salt stress response of EV control versus *VvARF6*-OE lines. For each genotype, more than 30 independent transgenic lines were irrigated with 150 mM NaCl for 21 days and typical lines are shown in (A) and (J), respectively. The following parameters were quantified: relative shoot growth rate (B, K), shoot fresh weight (C, L), shoot dry weight (D, M), root fresh weight (E, N), root dry weight (F, O), root activity (G, P), root malondialdehyde (MDA) content (H, Q), and chlorophyll content (I, R). Data represent means ± SD (n = 3 independent biological replicates). Asterisks above the bars indicate significant differences determined by Student's *t* test (*P* < 0.05).

To investigate the functional role of VvmiR167b in salt tolerance, we generated grapevine plants with roots overexpressing VvmiR167b (VvmiR167b-OE) using an *A. rhizogenes***-**mediated hairy root transformation system. Under salt stress conditions, grape plants carrying VvmiR167b-OE hairy roots exhibited superior overall growth compared to EV plants. Specifically, VvmiR167b-OE plants displayed a significantly higher relative shoot growth rate and increased shoot fresh weight (Fig. 7A–D), alongside enhanced root biomass and root activity (Fig. 7E–G). Furthermore, MDA content in roots was significantly reduced (Fig. 7H), while chlorophyll content was significantly elevated (Fig. 7I) in the overexpression lines. To further corroborate the function of VvmiR167b, we overexpressed it in *A. thaliana*. Seed germination assays showed that VvmiR167b-OE transgenic lines had a significantly higher germination rate than WT under salt stress (Fig. S7A and B). Consistent with this, salt-stressed VvmiR167b-OE seedlings exhibited significantly greater root length and fresh weight compared to WT seedlings (Fig. S7C–E). These results demonstrate that VvmiR167b enhances plant tolerance to salt stress.

Similarly, we examined the role of its target, *VvARF6*, in salt stress response. Grape plants carrying *VvARF6*-OE hairy roots displayed compromised growth under salt stress relative to EV controls. These plants showed a significantly reduced relative shoot growth rate and decreased shoot fresh weight (Fig. 7J–M), as well as diminished root biomass and root activity (Fig. 7N–P). Root MDA content was significantly elevated (Fig. 7Q), and chlorophyll content was significantly reduced (Fig. 7R) in *VvARF6*-OE lines. Ectopic overexpression of *VvARF6* in *Arabidopsis* similarly resulted in reduced salt tolerance. Salt-stressed *VvARF6*-OE seedlings had significantly shorter roots and lower fresh weight than WT seedlings (Fig. S8A–C). These results indicate that *VvARF6* negatively regulates plant salt tolerance, functionally opposing the effect of VvmiR167b.

## Discussion

U2A′ is evolutionarily conserved across eukaryotes including humans, plants, yeast, and nematodes [31]. Although spliceosomal components are increasingly implicated in plant salt stress responses [5, 32], the specific role of U2A′, a core component of the U2 snRNP, in plant abiotic stress tolerance has remained largely unexplored. Our study reveals that *VvU2A′* expression is dynamically induced by salt stress in grapevine (Fig. 1A). Furthermore, the promoter region of *VvU2A′* is enriched with diverse cis-acting elements responsive to various stresses and hormones (Fig. S1D), indicating its expression is modulated by complex environmental signaling networks. Mirroring findings in *C. deneoformans* JEC21 where U2A′ positively regulates stress tolerance [7], we found that overexpression of *VvU2A′* significantly improved salt tolerance in both grapevine and *Arabidopsis*. This enhanced tolerance was manifested by attenuated growth inhibition, maintained photosynthetic efficiency (e.g. Fv/Fm), elevated antioxidant enzyme activities, reduced oxidative damage (e.g. lower MDA and H₂O₂ levels), and improved ion homeostasis (e.g. lower Na^+^/K^+^ ratio) under salt stress. Conversely, silencing *VvU2A′* resulted in compromised salt tolerance phenotypes (Figs 3 and S2 and S3). Collectively, these results establish *VvU2A′* as a positive regulator of salt tolerance in grapevine and provide foundational insights into the potential conserved function of U2A*′* orthologues in stress adaptation across other plant species.

Previous studies in Drosophila and our prior work in grapevine have established that circRNA biogenesis depends on the spliceosome [15, 33]. However, whether spliceosomal activity modulates circRNA formation to influence plant stress tolerance remained unexplored. This study demonstrates that *VvU2A′* modulates the expression of hundreds of circRNAs under salt stress, representing, to our knowledge, the first investigation into the regulation of circRNA biogenesis by a spliceosomal factor under stress conditions in plants. U2A*′* plays a well-characterized core role in canonical pre-mRNA splicing as a structural scaffold within the U2 snRNP. It forms a heterodimer with U2B″ that specifically recognizes and stabilizes Stem-Loop IIa (SLIIa) of the U2 snRNA, thereby ensuring U2 snRNP assembly integrity and functional activity [34]. This U2A*′*/U2B*′′* complex is essential during the early stages of spliceosome assembly: the U2 snRNP must accurately bind the branch point sequence (BPS) of the intron, and the U2 snRNA conformation maintained by the U2A*′*/U2B*′′* heterodimer provides the structural basis for U2-BPS base-pairing [35]. We hypothesize that *VvU2A′* influences the efficiency of circRNA generation via back-splicing by modulating the binding capacity of the U2 snRNP to pre-mRNAs. Given that splice factors often act in concert through protein–protein interactions or in association with other regulatory factors to fine-tune splicing outcomes [36, 37], it is plausible that *VvU2A′* functions within a protein complex, potentially involving specific interactors, to precisely dictate whether particular grape circRNAs are up- or down-regulated in response to salt stress. The parental genes of these circRNAs were enriched in GO terms, such as ‘negative regulation of organ growth’, ‘positive regulation of leaf senescence’, ‘negative regulation of cell proliferation’, ‘negative regulation of mitotic sister chromatid segregation’, and ‘negative regulation of protein localization to kinetochore’, as well as in KEGG pathways, including ‘linoleic acid metabolism’, ‘valine, leucine and isoleucine biosynthesis’, and ‘lysine biosynthesis’. These enrichments suggest that the identified circRNAs may influence cell proliferation and growth under salt stress by modulating amino acid biosynthesis and related metabolic processes.

To date, relatively few circRNAs with clearly defined functions have been reported in plants [11]. In this study, we investigated the function of *VvcircHMA1*, a circRNA negatively regulated by *VvU2A′*, during salt stress. Unlike *VvcircSIZ1* and *VvcircABH* that positively regulate salt tolerance [15, 38], overexpression of *VvcircHMA1* significantly enhanced salt sensitivity in grape calli, hairy root plants, and *N. benthamiana* (Figs 5 and S5). This characterization of *VvcircHMA1* expands the repertoire of functionally defined, stress-responsive circRNAs in grapevine. Critically, the contrasting salt stress phenotypes observed upon *VvU2A′* overexpression (enhanced tolerance) versus *VvcircHMA1* overexpression (enhanced sensitivity) strongly support a regulatory pathway wherein *VvU2A′* promotes salt tolerance, at least in part, by suppressing the accumulation of the salt-sensitizing circRNA *VvcircHMA1*.

The competitive endogenous RNA (ceRNA) mechanism is the most prominent functional paradigm for circRNAs in animals [22]. Although plant circRNAs are commonly predicted as potential ceRNAs, this role remains largely supported by bioinformatic predictions, with only a handful of cases experimentally validated to date [13]. For instance, our prior study demonstrated that grapevine *Vv-circSIZ1* enhances salt tolerance by sequestering VvmiR3631 to upregulate its target *VvVHac1* [15]. Similarly, a recent study in rice identified *circANK* as a ceRNA that sequesters miR398b to modulate its target genes, consequently negatively regulating resistance to bacterial blight [14]. In the present study, we provide integrated bioinformatic and experimental evidence that *VvcircHMA1* functions as a ceRNA through binding VvmiR167b, thereby attenuating its cleavage activity on the target gene *VvARF6* (Fig. 6). Functionally, we found that overexpression of VvmiR167b enhanced salt tolerance in both grapevine and *Arabidopsis* (Fig. 7A–I). Notably, this finding contrasts with a previous report showing that silencing miR167 increased salt stress tolerance (specifically, improved germination rate) in *N. benthamiana* [39]. This discrepancy may be attributed to several factors. First, ARF transcription factors exhibit considerable functional diversity [40]. Although the miR167 targeting sequence is conserved, *VvARF6* and its *N. benthamiana* homologs may regulate distinct sets of downstream genes. Second, and more critically, the experimental systems employed—whole grapevine plants in this study versus *in vitro* organ cultures in *N*. *benthamiana*—differ substantially in stress perception and overall physiological context, which could significantly influence the resulting phenotypic outcomes. Furthermore, numerous studies have reported that the same miRNA, or even different members of the same miRNA family within a species, can exert opposite effects on salt tolerance, highlighting the complexity and diversity of miRNA regulatory networks in plants [41].

As a target gene for miR167, *ARF6* participates in multiple plant developmental processes, such as fruit development, adventitious root formation, floral bud growth, and leaf development [42, 43]. For instance, *PmARF6* negatively regulates flowering and leaf growth speed in *Pinus massoniana* [44]. Interestingly, in our study, overexpression of *VvARF6* did not alter the growth phenotype under normal conditions in transgenic grapevine or *Arabidopsis*, suggesting potential functional diversification of *ARF6* orthologues during evolution. Crucially, however, under salt stress, *VvARF6*-OE significantly suppressed shoot and root growth in grapevine (Fig. 7J–R), establishing *VvARF6* as a negative regulator of salt tolerance in this species. Whether *ARF6* orthologs consistently confer negative regulation of salt tolerance across other plant species warrants further investigation. Collectively, the salt tolerance-promoting phenotype of VvmiR167b overexpression, the salt sensitivity-conferring phenotype of *VvARF6* overexpression, and the enhanced salt sensitivity observed upon *VvcircHMA1* overexpression form a coherent and mutually reinforcing regulatory axis. These findings establish the *VvcircHMA1***-**VvmiR167b**-***VvARF6* pathway as compelling new functional evidence supporting the ceRNA activity of circRNAs in plants.

**Figure 8:**
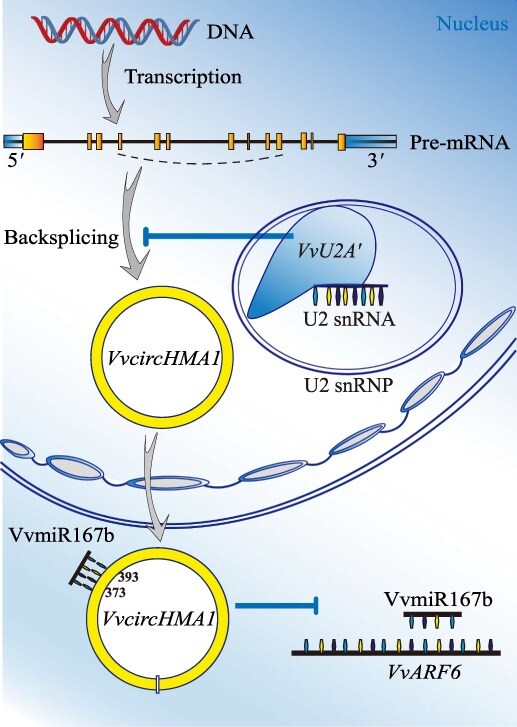
Biosynthesis of *VvcircHMA1* and its role in salt stress regulation. Salt stress upregulates *VvU2A′*, which suppresses *VvcircHMA1* expression. *VvcircHMA1* functions as a competitive endogenous RNA (ceRNA), sequestering VvmiR167b to modulate *VvARF6* expression, thereby regulating salt tolerance in grapevine. The nucleotide positions 373–393 in the *VvcircHMA1* sequence indicate the binding site for VvmiR167b. Arrows indicate activation or a positive regulatory relationship; blunt-ended lines represent inhibition or a negative regulatory relationship.

In summary, this study elucidates a novel mechanism by which the U2 snRNP core protein *VvU2A′* positively regulates salt tolerance in grapevine (*V. vinifera*) (Fig. 8). Salt stress induces *VvU2A′* expression, which suppresses *VvcircHMA1* biogenesis. Reduced *VvcircHMA1* levels attenuate sequestration of VvmiR167b, leading to increased free VvmiR167b that enhances cleavage of its target *VvARF6* mRNA. The resultant decrease in *VvARF6* expression promotes salt stress tolerance in grapevine. The discovery of this *VvU2A′***-***VvcircHMA1***-**VvmiR167b**-***VvARF6* regulatory pathway unveils a molecular pathway through which spliceosomal components mediate stress adaptation by modulating a circRNA–ceRNA network. Our findings provide new insights into the complex plant stress response network and offer a theoretical foundation and potential targets for molecular breeding of salt-tolerant grapevines. Future investigations could explore the target gene network downstream of *VvARF6* and the conservation of this pathway across plant species and stress types.

## Materials and methods

### Plant materials

*V. vinifera* cv. Cabernet Sauvignon, *N. benthamiana*, and *A. thaliana* were used. During winter pruning, 1-year-old canes from healthy 6-year-old grapevines were collected and stored in sand for *A. rhizogenes***-**mediated transformation. Six-week-old *N. benthamiana* and *A. thaliana* plants grown in climate chambers (25–28°C, 16-hour photoperiod) were used for *A. tumefaciens***-**mediated transient transformation assays.

### CircRNA-Seq

Total RNA for circRNA-Seq was extracted from wild-type and *VvU2A′*-overexpressing grapevine calli using the mirVana™ miRNA Isolation Kit (Ambion, Austin, TX, USA). Ribosomal RNA was removed from 2 μg total RNA using Ribo-Zero Plant (RS-122-2401; Illumina, San Diego, CA, USA) with TruSeq Stranded Total RNA, followed by linear RNA depletion with RNase R (Epicenter, San Diego, CA, USA). Sequencing libraries were prepared as previously described [45] and sequenced on an Illumina HiSeq™ 2500 system. rRNA reads and low-quality reads were filtered from raw data using SortMeRNA and Trimmomatic, respectively. CIRI2 was employed for circRNA identification, with expression levels quantified by RPM (back-spliced junction reads per million mapped reads). Differentially expressed circRNAs were identified using the thresholds: |fold-change| > 2 and *P* value <0.05.

### Bioinformatic analysis

The grapevine reference genome sequence (PN40024 12X.v2), including DNA sequences, protein (amino acid) sequences, and annotation files, was retrieved from theCRIBI Database [46]. Basic physicochemical properties and genomic features of *VvU2A′* (e.g. chromosome location, number of introns/exons, coding sequence (CDS) length) were extracted from the annotation data. Phylogenetic analysis was performed using the Molecular Evolutionary Genetics Analysis (MEGA) software (version X 10.1). Multiple sequence alignments of U2A′ amino acid sequences from diverse plant species were constructed, and a phylogenetic tree was inferred using the neighbor-joining (NJ) method with [Specify Bootstrap Value, 1000] bootstrap replicates. Putative cis-acting regulatory elements within the promoter region (specify region, 1500 bp upstream of the transcription start site) of U2A′ were predicted using the PlantCARE online tool. The potential binding sites of VvmiR167b on *VvcircHMA1* and *VvARF6* were predicted using the psRNATarget software using default parameters [47].

### RT-PCR and RT-qPCR analysis

Total RNA was extracted from plant tissues using the Plant Total RNA Extraction Kit (Tiangen Biotech, DP441). First-strand cDNA was synthesized from total RNA a mixture of oligo(dT) and random hexamer primers. Linear mRNAs and circRNAs were analyzed by conventional PCR and RT-qPCR using convergent and divergent primers for qualitative detection and quantitative measurement of their expression, respectively. The back-splice junctions of circRNAs were further verified by Sanger sequencing of the PCR amplicons. For mature miRNA expression analysis, small RNAs were isolated using the miRNA Isolation Kit (Tiangen Biotech, DP504) and reverse-transcribed with miRNA-specific stem**-**loop RT primers. Quantitative PCR (qPCR) was conducted using TB Green® Premix Ex Taq™ II (Takara, Japan) on a CFX96 Real-Time PCR Detection System (Bio-Rad, USA). *VvActin* served as the reference gene, and relative expression levels were calculated via the 2^−ΔΔCt^ method. All primer sequences used for PCR and qPCR are listed in Table S1.

### Expression vector construction

Specific primers were designed using the Vazyme CE Design Platform, with all oligonucleotides synthesized by Sangon Biotech (Shanghai, China) (Table S1). Target gene fragments were amplified from grape leaf cDNA templates via high-fidelity DNA polymerase-mediated PCR (Takara Bio Inc., Japan). Amplified products were size-verified by agarose gel electrophoresis, followed by gel excision and purification. Purified fragments were ligated into linearized vectors (digested with corresponding restriction enzymes) using homologous recombination cloning to construct recombinant expression vectors.

For *VvcircHMA1^mu^*, site-directed mutagenesis was performed using the Mut-Express® II Fast Mutagenesis Kit (Vazyme, Nanjing, China), with mutant fragments validated by Sanger sequencing to confirm exclusive introduction of intended mutations. Sequence-verified recombinant plasmids and EV controls were transformed into *A. tumefaciens* GV3101 (pSoup-p19) or *A. rhizogenes* MSU440 strains via the freeze–thaw method for subsequent plant transformation.

### Transient transformation assay in *N. benthamiana*

*A. tumefaciens* strains (GV3101) harboring the recombinant plasmid of interest were cultured overnight, harvested by centrifugation, and resuspended in infiltration buffer (10 mM MgCl₂, 10 mM MES pH 5.6, supplemented with 100 μM acetosyringone). The optical density of the bacterial suspension was adjusted to OD_600_ = 0.6 using a UV–Vis spectrophotometer. The suspension was incubated at 28°C for 2 to 3 hours without shaking. *N. benthamiana* leaves were infiltrated with the bacterial suspension using a needleless syringe. For subcellular localization analysis, leaves expressing the GFP-fusion construct were examined using a super-resolution laser scanning confocal microscope (LSCM; Zeiss, Oberkochen, Germany) 3 days postinfiltration (dpi), and images were captured. For luciferase (LUC) reporter assays, infiltrated leaves were sprayed with 100 mM D-luciferin (potassium salt) solution and kept in complete darkness for 5 minutes prior to imaging. LUC activity was visualized and quantified using an IVIS Lumina II *in vivo* imaging system (Xenogen, Alameda, CA, USA).

### Stable genetic transformation

*Arabidopsis thaliana* was transformed using the floral dip method mediated by *A. tumefaciens* strain GV3101 [48]. *N. benthamiana* was transformed via the leaf disk method using *A. tumefaciens* strain GV3101 [49]. Homozygous lines were selected for phenotypic analysis. Transgenic grape calli were generated through *A. tumefaciens* (strain LBA4404)-mediated transformation [50].

### Hairy root transformation in grapevine mediated by *A. rhizogenes*

Hairy root transformation in grapevine was performed essentially as described previously [51] with minor modifications. Dormant grapevine cuttings stored in sand were retrieved in spring and sectioned into approximately 30 cm segments, each containing a bud. The recombinant plasmid-carrying *A. rhizogenes* strain MSU440 was cultured overnight at 28°C in Lysogeny Broth (LB) medium supplemented with kanamycin (50 mg∙L^−1^) and rifampicin (50 mg∙L^−1^). Bacterial cells were harvested by centrifugation, resuspended in an infiltration buffer (containing 100 μM acetosyringone, 10 mM MES, 10 mM MgCl₂, pH 5.8), and adjusted to an optical density at 600 nm (OD_600_) of 0.7. The basal ends of the grapevine cuttings were immersed in the bacterial suspension and incubated overnight (~16 hours) at room temperature. Subsequently, the inoculated cuttings were transferred to a sand bed and cultivated until the three-leaf stage. Transgenic plants were then transplanted into pots containing a mixture of nursery substrate and river sand and grown until the 10-leaf stage for subsequent salt stress treatments. Each individual plant was considered one biological replicate, and all physiological assays were performed with at least three independent biological replicates.

### Salt stress treatments and salt tolerance analysis

Seed germination assay: Surface-sterilized seeds of *N. benthamiana* or *A. thaliana* were sown on half-strength Murashige and Skoog (½ MS) medium supplemented with different concentrations of NaCl (100, 125, or 150 mM). Plates were incubated under standard growth conditions for 10 days, after which the germination rate was quantified.

Seedling growth assay on plates: 7-day-old seedlings of *N. tabacum* or *A. thaliana* were transferred onto ½ MS medium containing various NaCl concentrations (100, 125, 150, or 200 mM). After 8 days of further growth under standard conditions, phenotypes were documented, and primary root length and whole-plant fresh weight (FW) were measured.

Soil-grown plant assay: 3-week-old potted plants of *N. tabacum* or *A. thaliana* were subjected to salt stress by irrigation with either 100 mM or 150 mM NaCl solution every 3 days, using water irrigation as the control. Plant growth phenotypes were observed and photographed 10 days after the initiation of treatment.

Grape callus stress assay: uniformly sized callus pieces derived from wild-type (WT) and transgenic grape lines were inoculated onto B5 solid medium supplemented with either 0 mM (control) or 100 mM NaCl. Growth status was photographed and callus fresh weight was recorded after 3 weeks of culture.

Grape plant stress assay: transgenic grape plants harboring engineered hairy roots were irrigated with 150 mM NaCl solution when they reached approximately 20 cm in height. Following 30 days of treatment, the overall plant phenotype was photographed, and key physiological and biochemical indices were measured.

### Determination of plant physiological and biochemical indices

Chlorophyll content was extracted with ethanol and quantified spectrophotometrically. Root activity was measured using triphenyltetrazolium chloride (TTC) reduction assays. Sodium and potassium ion concentrations in roots, stems, and leaves were determined by flame photometry.

Leaf REC was measured using a conductivity meter (Mettler Toledo, Switzerland). Chlorophyll fluorescence parameters in the fourth-node leaves of grapevine were recorded with a FMS-2 portable pulse-modulated fluorometer (Hansatech, UK).

Superoxide dismutase and CAT activities were assayed using commercial kits (Griess Biotechnology, Suzhou, China). MDA content in roots was quantified with an MDA detection kit (Griess Biotechnology).

### NBT and DAB staining

For NBT staining, *Arabidopsis* leaves were immersed in 100 ml of 0.5 mg·ml^−1^ NBT solution (10 mM potassium phosphate buffer, pH 7.8) in a 500-ml beaker. After vacuum infiltration, samples were incubated in darkness at room temperature for 1 h. Leaves were then decolorized by boiling in 95% ethanol until complete chlorophyll removal and photographed.

For DAB staining, leaves were submerged in 100 ml of 1 mg·ml^−1^ DAB solution (50 mM Tris–HCl, pH 3.8) in a 500-ml beaker. Following vacuum infiltration, samples were maintained in darkness at 28°C for 14 hours. The staining solution was discarded, and leaves were boiled in 95% ethanol until fully decolorized before imaging.

### Statistical analysis

Statistical analyses were performed using SPSS 22.0 (IBM., Inc., Armonk, NY, USA). Differences between two groups were assessed by a paired two-tailed Student's *t* test, while comparisons among multiple groups were performed by one-way ANOVA followed by Duncan’s test. Statistical significance was defined at *P* < 0.05.

## Supplementary Material

Web_Material_uhaf355

## Data Availability

Supplementary information accompanies the manuscript in the supplemental tables and files provided. The raw circRNA sequencing data generated in this study have been deposited in the Genome Sequence Archive (GSA; http://bigd.big.ac.cn/gsa/) under accession number PRJCA049440.
