# Supplementary material for: VvU2A′-mediated circRNA biogenesis confers salt tolerance in grapevine via the VvcircHMA1-VvmiR167b-VvARF6 pathway
Source: Hortic Res. 2025 Dec 22;13(4):uhaf355. doi: 10.1093/hr/uhaf355 (PMC13091397; doi:10.1093/hr/uhaf355)
Supplement: Web_Material_uhaf355 [file web_material_uhaf355.zip › Supplementary figures.docx]

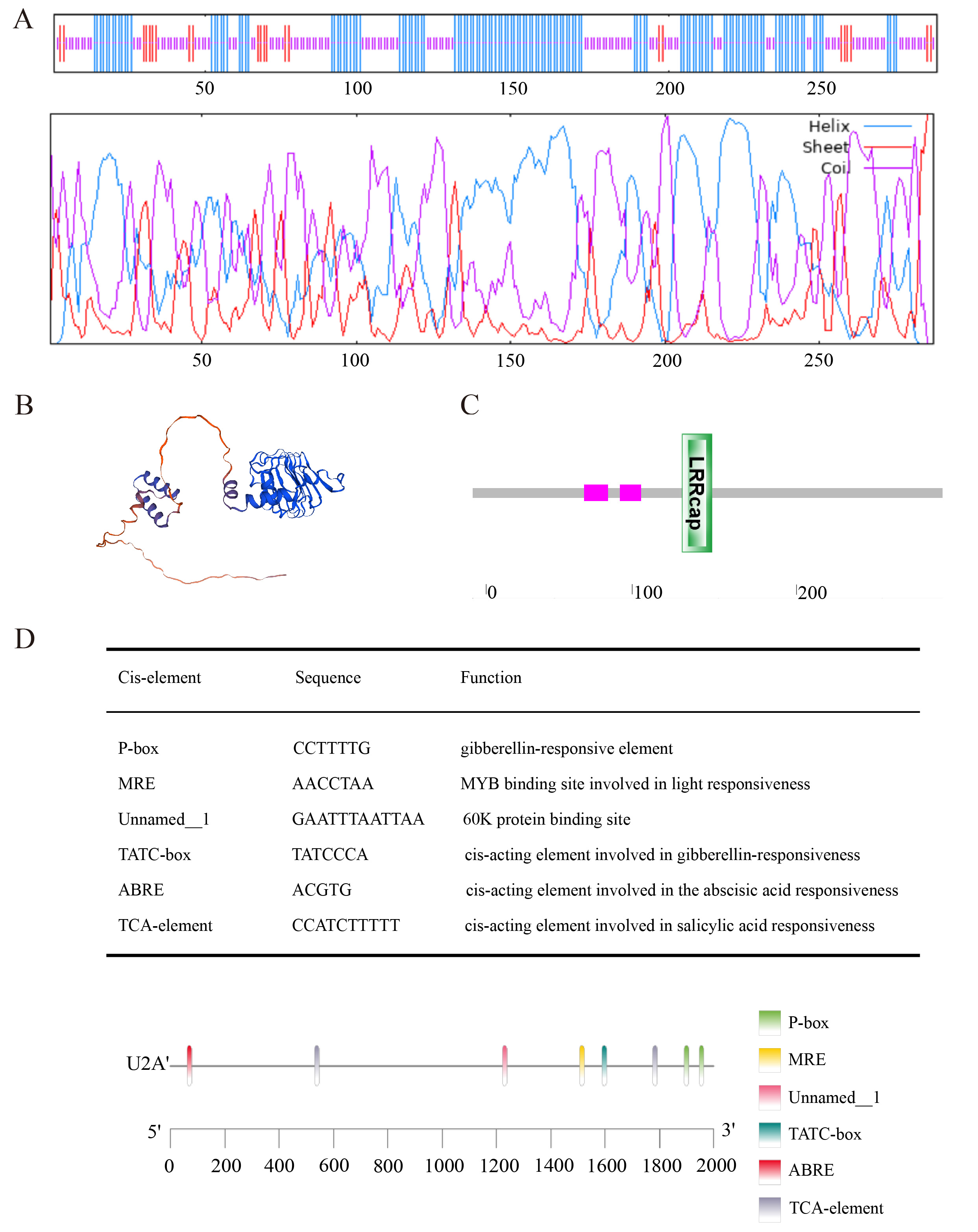


**Figure S1 Characterization of *VvU2A'* in grapevine.** (A) Secondary structure of *VvU2A'* protein predicted by SOPMA (α-helix 45.99%, extended strand 8.01%, random coil 45.99%). (B) Tertiary structure of *VvU2A'* predicted by SWISS-MODEL homology modeling. (C) Conserved domains in *VvU2A'* identified by SMART database. (D) Cis-acting elements in the *VvU2A'* promoter region (2000 bp) predicted using PlantCARE. Key elements: ABRE (abscisic acid-responsive, red), TATC-box (gibberellin-responsive, dark green), TCA-element (salicylic acid-responsive, gray), MRE (light-responsive, yellow).


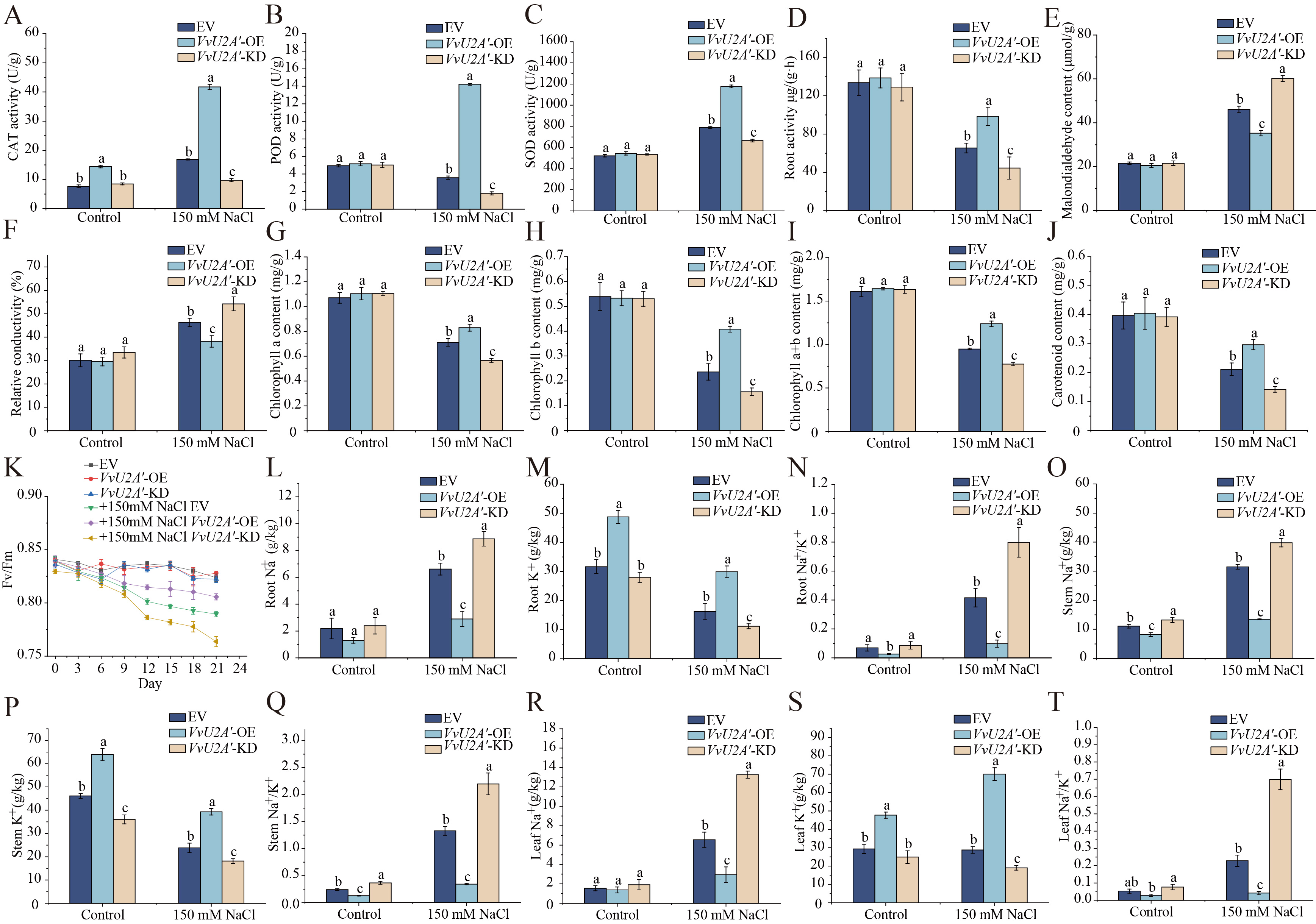


**Figure S2 Effects of *VvU2A'* overexpression/silencing on physiological and biochemical parameters in grapevine under salt stress.** Data are expressed as mean ± standard error (SD) (n=3 independent biological replicates). Different letters above the bars indicate signiﬁcantly different values (*P* < 0.05) calculated using one-way ANOVA, followed by Duncan's multiple range test.


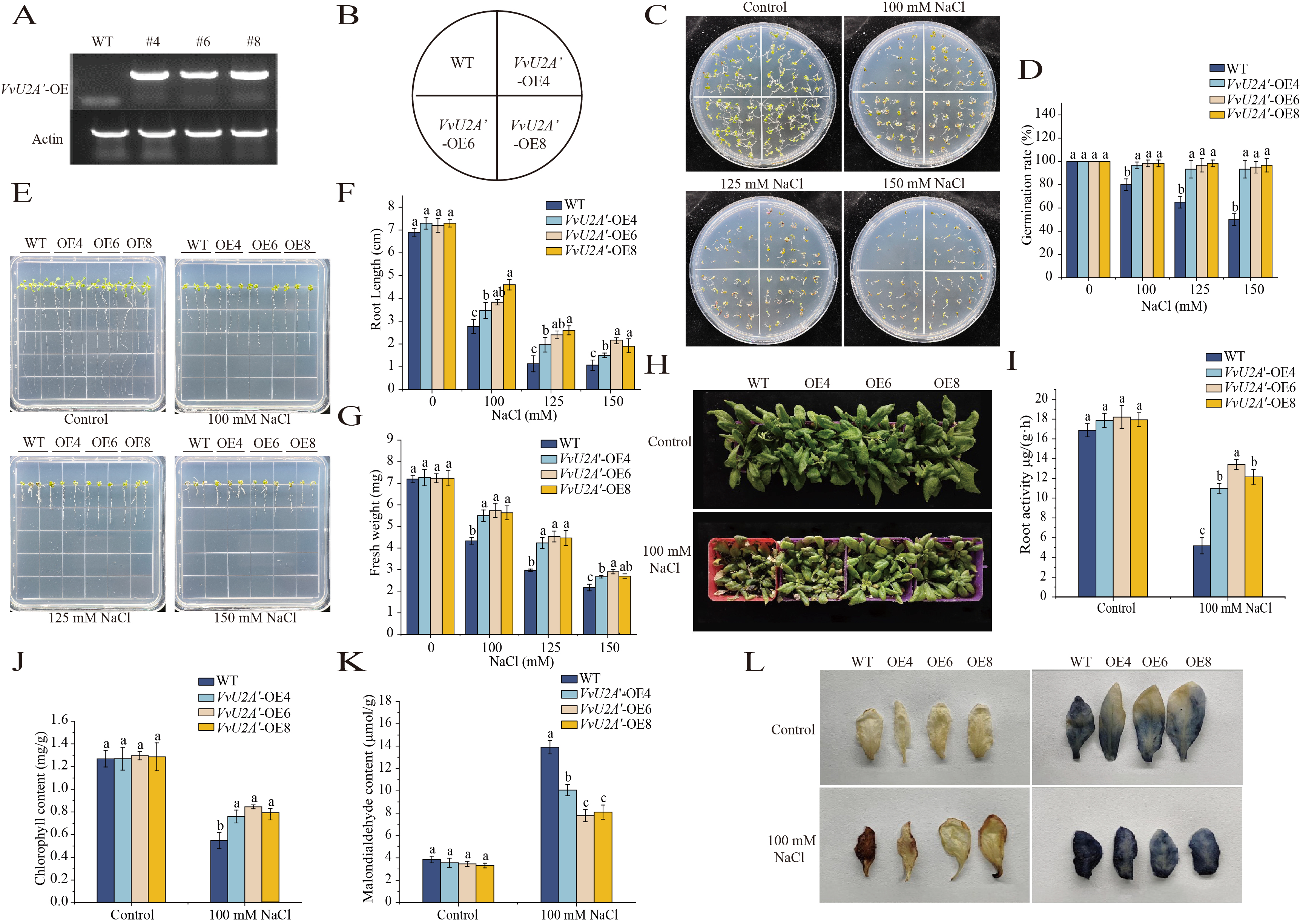


**Figure S3 Overexpression of *VvU2A'* enhances salt tolerance in *Arabidopsis*.** (A) RT-PCR analysis confirming the expression of the *VvU2A'* overexpression construct in transgenic *Arabidopsis* lines using gene-specific and vector-specific primers. (B-D) Effects of *VvU2A'* overexpression on seed germination under salt stress. (C) Phenotype. (D) Germination rate. (E-G) Effect of *VvU2A'* overexpression on salt tolerance of seedlings grown on MS medium. (E) Growth phenotypes under salt stress; (F) Primary root length; (G) Plant fresh weight. (H-L) Salt tolerance of soil-grown *VvU2A'*-OE *Arabidopsis*. Growth phenotype (H); Root activity (I); Chlorophyll content (J); MDA content (K); Histochemical detection of H₂O₂ and O₂⁻ accumulation by 3,3'-diaminobenzidine (DAB) and nitroblue tetrazolium (NBT) staining, respectively (L). Data represent mean ± SD (n=3 independent biological replicates). Different letters above the bars indicate signiﬁcantly different values (*P* < 0.05) calculated using one-way ANOVA, followed by Duncan's multiple range test.


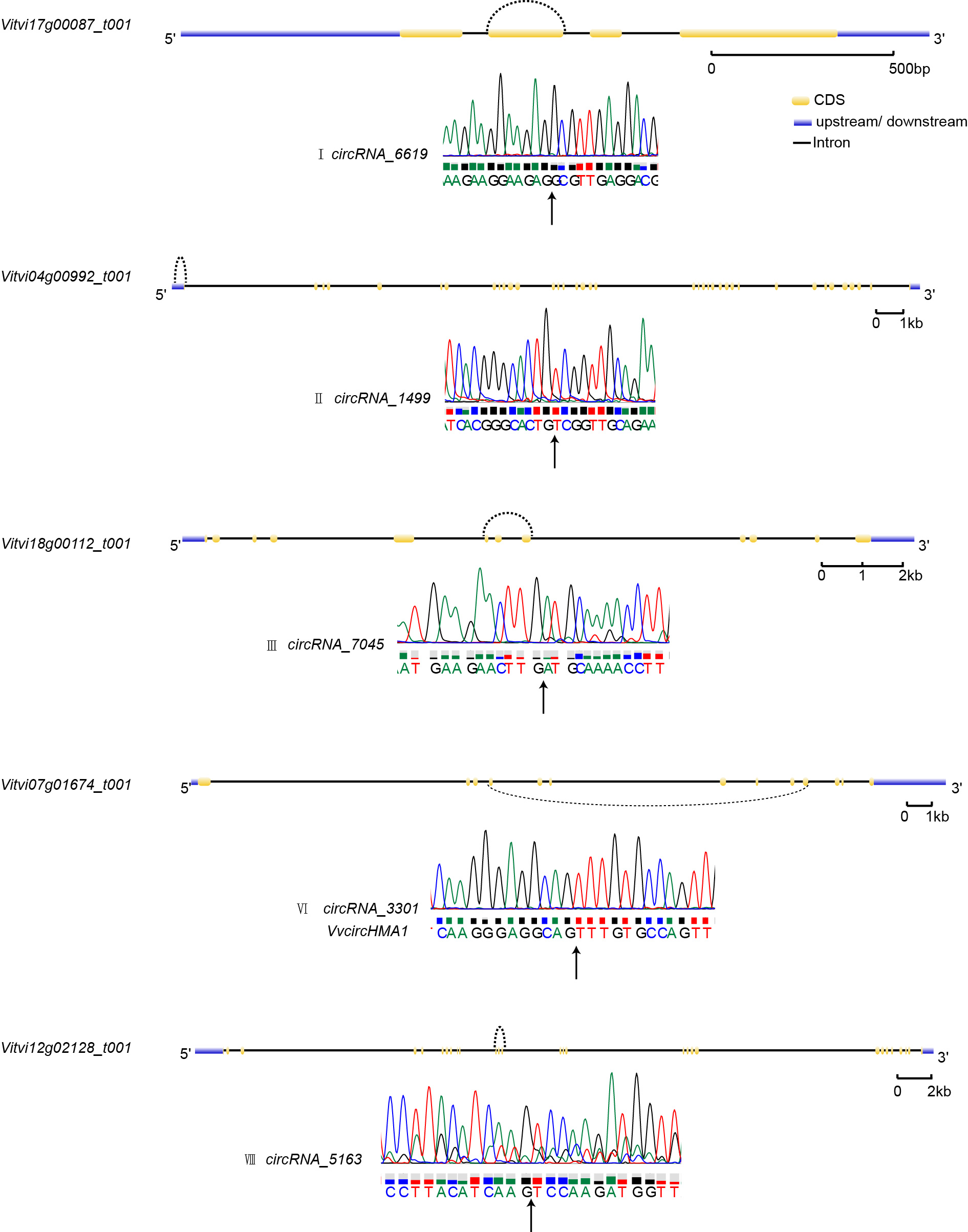


**Figure S4 Ampliﬁcation and validation of five grape circRNAs by PCR using divergent primers.** The upper diagram represents the unique number of circRNAs and their host simulation gene structure. In the bottom, the Sanger sequencing results of PCR products ampliﬁed from cDNA with a pair of divergent primers.


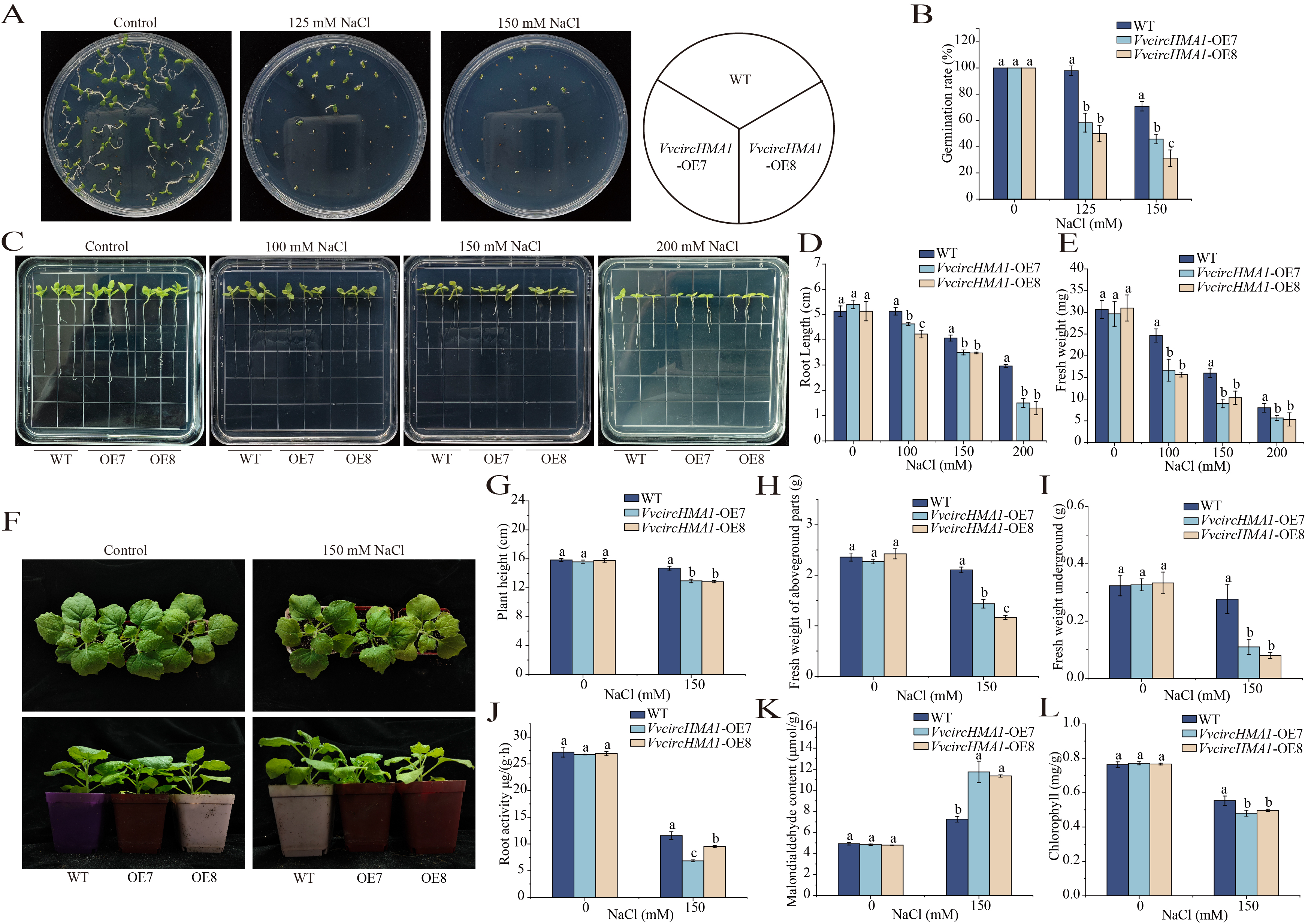


**Figure S5 Overexpression of *VvcircHMA1* negatively regulates salt tolerance in *Nicotiana benthamiana*.** (A and B) Effects of *VvcircHMA1* overexpression on *Nicotiana benthamiana* seed germination under salt stress. (A) Germination phenotypes. (B) Germination rate. (C-E) Effect of *VvcircHMA1* overexpression on salt tolerance of *Nicotiana benthamiana* seedlings grown on MS medium. (C) Growth phenotypes; (D) Root length; (E) Seedling fresh weight. (F-L) Effect of *VvcircHMA1* overexpression on salt tolerance in soil-grown *Nicotiana benthamiana* plants. (F) Growth phenotypes; (G) Plant height; (H) Shoot fresh weight; (I) Root fresh weight; (J) Root activity; (K) Root malondialdehyde (MDA) content; (L) Chlorophyll content. Data represent mean ± SD (n=3). Different letters above the bars indicate signiﬁcantly different values (*P* < 0.05) calculated using one-way ANOVA, followed by Duncan's multiple range test.


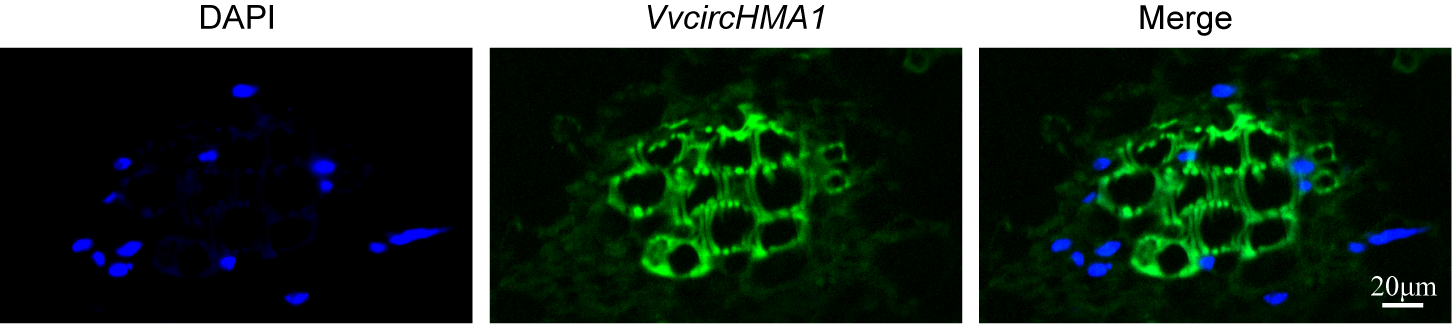


**Figure S6.** RNA in situ hybridization analysis of *VvcircHMA1* in *Nicotiana benthamiana* leaves. The antisense probe was designed to target the back splice junction site of *VvcircHMA1*. Nuclei were stained with 40 ,6-diamidino-2-phenylindole (DAPI; blue). *VvcircHMA1* appeared green, and overlapping expression was indicated in the merged image.


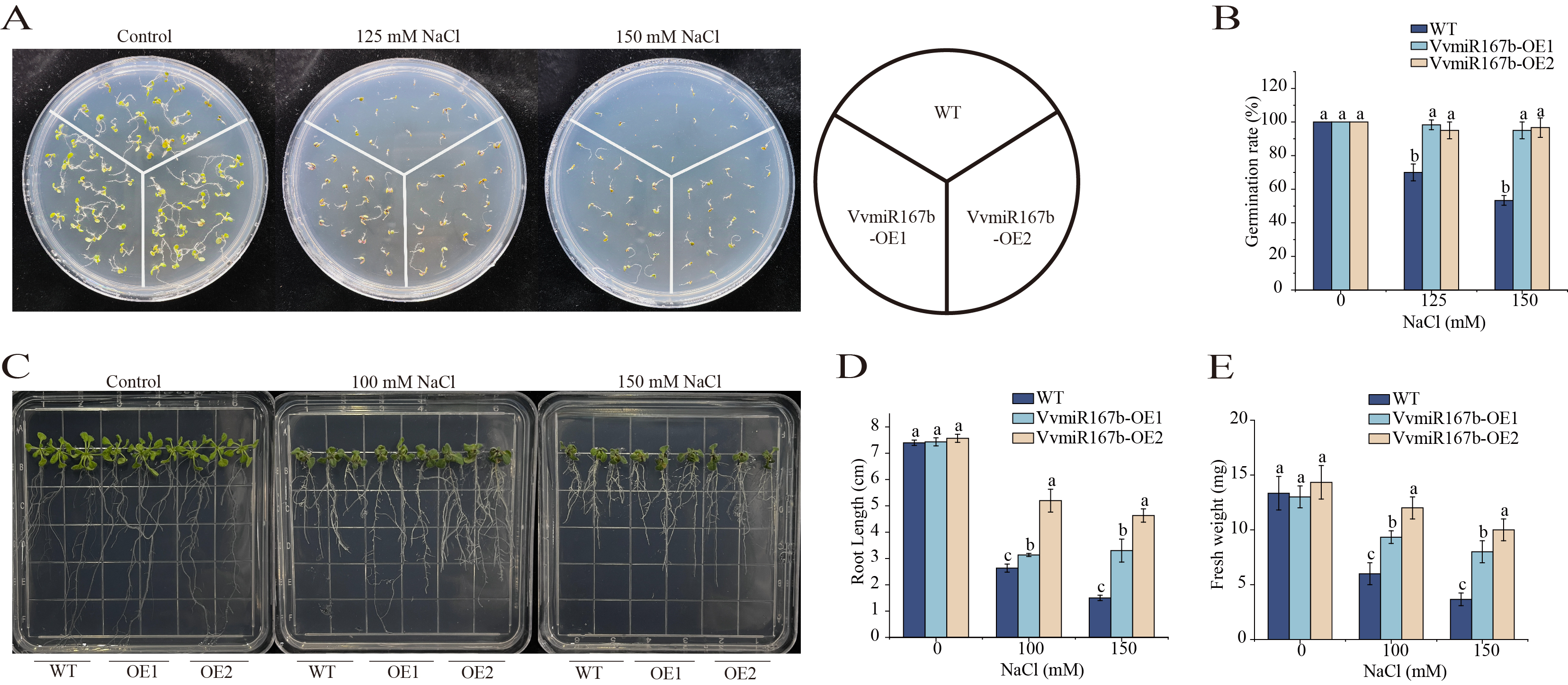


**Figure S7 Overexpression of VvmiR167b enhances salt tolerance in *Arabidopsis*.** (A and B) Effect of VvmiR167b overexpression on *Arabidopsis* seed germination under salt stress. (A) Germination phenotypes. (B) Germination rates. (C-E) Effects of VvmiR167b overexpression on salt tolerance of *Arabidopsis* seedlings grown on MS medium. (C) Growth phenotype. (D) Root length. (E) Fresh weight. Data represent mean ± SD (n=3). Different letters above the bars indicate signiﬁcantly different values (*P* < 0.05) calculated using one-way ANOVA, followed by Duncan's multiple range test.


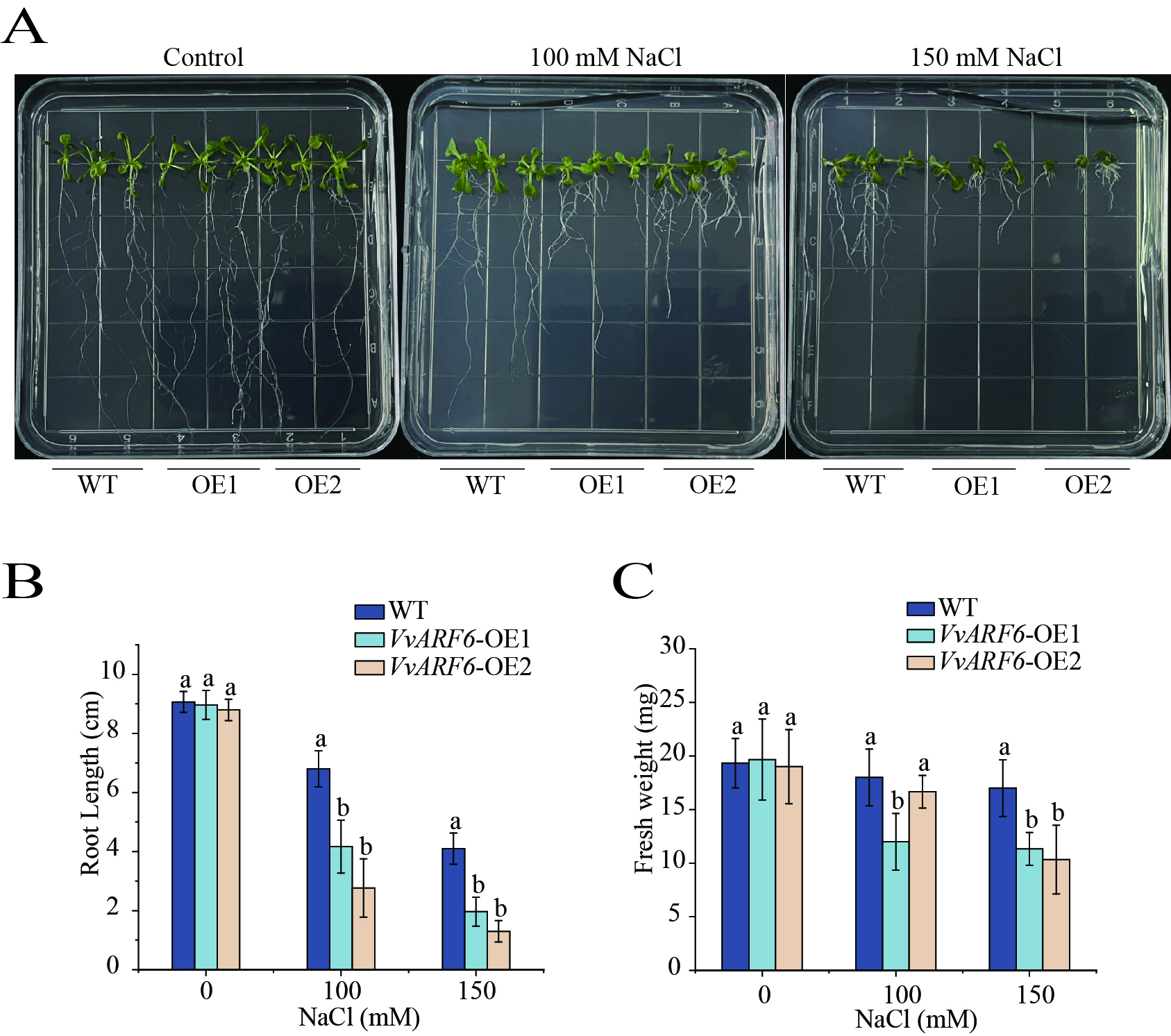


**Figure S8 Overexpression of *VvARF6* suppresses salt tolerance in *Arabidopsis*.** (A-C) Effect of *VvARF6* overexpression on salt tolerance in *Arabidopsis* grown on MS medium. (A) Growth phenotype. (B) Root length. (C) Fresh weight. Data represent mean ± SD (n=3). Different letters above the bars indicate signiﬁcantly different values (*P* < 0.05) calculated using one-way ANOVA, followed by Duncan's multiple range test.
