# Supplementary material for: VvU2A′-mediated circRNA biogenesis confers salt tolerance in grapevine via the VvcircHMA1-VvmiR167b-VvARF6 pathway
Source: Hortic Res. 2025 Dec 22;13(4):uhaf355. doi: 10.1093/hr/uhaf355 (PMC13091397; doi:10.1093/hr/uhaf355)
Supplement: Web_Material_uhaf355 [file web_material_uhaf355.zip › Supplementary table.docx]

**Table S1 Primer sequences used in this study.**

| Primer Name | Sequence (5ʹ→3ʹ) |
| --- | --- |
| *VvU2A'*-qPCR-F | GGCTCACTGCAGACCTGATT |
| *VvU2A'*-qPCR-R | CTGTAGCGCCCAAGTTCTCA |
| *VvU2A'*-gfp-F | accagtctctctctcaagcttATGGTGAGGCTCACTGCAGACC |
| *VvU2A'*-gfp-R | gcccttgctcaccatggatccTTCCTGTTCCATTGGTGCCA |
| *VvU2A'* KD-F | accagtctctctctcaagcttATGGTGAGGCTCACTGCAGACC |
| *VvU2A'* KD-R | CTAGCGTGTGTAATTTTGGCAAGT |
| *VvU2A'* KD-F | gccaaaattacacacgctagACTAGTAAAATACAACACATGTTAATTGATACA |
| *VvU2A'* KD-R | cacgctagGTTTATAGGTGCTTTAAAGTACTCAAATTG |
| *VvU2A'* KD-F | gcacctataaacCTAGCGTGTGTAATTTTGGCAAGT |
| *VvU2A'* KD-R | gcccttgctcaccatggatccATGGTGAGGCTCACTGCAGACC |
| *circRNA_6619*-F | GTTCAGAGGAAGAACCACAGACA |
| *circRNA_6619*-R | CTTTCAATGGATCGGCCTTT |
| *circRNA_1499*-F | TATATCCGTGATGTTGGTTTTGGT |
| *circRNA_1499*-R | ATACCCACAGCGGTCCCATA |
| *circRNA_7045*-F | TGGTAATGTGAATGCGCCATA |
| *circRNA_7045*-R | GCCATCACTGAAACCTCTGTCA |
| *circRNA_3301*-F | GAGTTGCAAATGCTGTGGGTATC |
| *circRNA_3301*-R | CCTTTCCCCAACTGTCCTCTCT |
| *circRNA_5163*-F | GTGATAAATCCTCTACATCCAACTTGAA |
| *circRNA_5163*-R | TGCACCAACCTTCGTCTTGA |
| *VvcircHMA1*-OE-F | accagtctctctctcaagcttTTTGTGCCAGTTGATTGTGAAGTT |
| *VvcircHMA1*-OE-R | gctcctgcagctcgaggatccCCTCCCTTGATATACTCTTCACATGA |
| VvmiR167b-OE-F | accagtctctctctctcaagcttAGTATGCCTGGAACAAGTAAAATTGA |
| VvmiR167b-OE-R | gctcctgcagctcgaggatccGAGCCAAAATCAAAGAGAAGAAGAT |
| *Vvactin*-F | GAGATTCCGTTGTCCAGAAGTC |
| *Vvactin*-R | CAATGTTGCCATAGAGGTCCTT |
| *VvARF6*-800Luc-F | agatcgccgtgtaattctagaATGAGGTTGTCTCCTGCTGGG |
| *VvARF6*-800Luc-R | agcgaattcactagtggatccGTAGTCAAGAGACCCCACAGATGTG |
